# Tuning of Proanthocyanidin Extract’s Composition through Quaternary Eutectic Solvents Extraction

**DOI:** 10.3390/antiox9111124

**Published:** 2020-11-13

**Authors:** Rodrigo T. Neto, Sónia A. O. Santos, Joana Oliveira, Armando J. D. Silvestre

**Affiliations:** 1 CICECO—Aveiro Institute of Materials, Chemistry Department, University of Aveiro, Campus de Santiago, 3810-193 Aveiro, Portugal; rtneto@ua.pt (R.T.N.); santos.sonia@ua.pt (S.A.O.S.); 2 REQUIMTE—Laboratório Associado para a Química Verde, Departamento de Química e Bioquímica, Faculdade de Ciências, Universidade do Porto, Rua do Campo Alegre, 687, 4169-007 Porto, Portugal; jsoliveira@fc.up.pt

**Keywords:** proanthocyanidin, eutectic solvents, extraction optimization, degree of polymerization, galloylation percentage

## Abstract

Currently available proanthocyanidins extraction methods rely on dedicated crops and have low specificity and yield which limits their industrial application. Consequently, the development of novel methodologies and the use of sustainable sources is of great importance. Eutectic solvents have been proposed has good alternatives for conventional solvents due to their low price, easiness of preparation, biocompatibility and ability of being custom made to a specific application. Herein the effective extraction of proanthocyanidins from grape pomace and the possibility of tuning the extract’s characteristics such as mean degree of polymerization and galloylation percentage is explored by means of varying the composition of a quaternary eutectic solvent composed by choline chloride, glycerol, ethanol and water. It was found that mean degree of polymerization values can vary from 6.0 to 7.37 and galloylation percentage can vary from 32.5% to 47.1% while maintaining extraction yield above 72.2 mg of proanthocyanidins per g of biomass. Furthermore, the increase of temperature up to 100 °C has showed a significant effect on the extraction yield being possible to increase it by 238% when compared to the conventional extraction method.

## 1. Introduction

**Figure 1:**
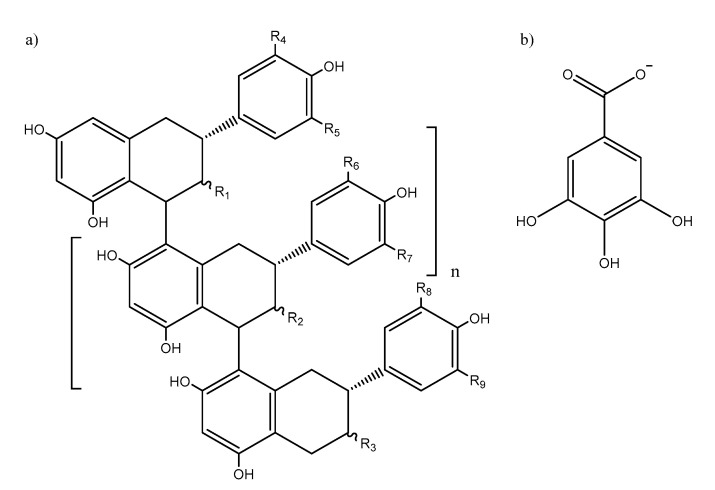
General molecular structure of (**a**) B-type proanthocyanidins (R1, R2, R3 = OH or (**b**) gallate units; R4, R5, R6, R7, R8, R9 = H or OH).

Proanthocyanidins (PACs), also known as condensed tannins, are secondary metabolites ubiquitous to all plant kingdom [1]. PACs are polymeric phenolic compounds comprising of flavan-3-ols monomers such as catechin and its derivatives (Figure 1). PACs are believed to play essentially two roles in plants, namely, as defense against microbial pathogens [2,3] and as deterrents against herbivory [4,5]. The mechanism by which PACs are able to achieve these functions come from their complexation ability of metal ions in the former case [6] and protein aggregation [7] and enzyme inhibition [8] in the latter.

Currently, PACs are mostly used in the production of high-end leather [9] and wood agglomerates [10] as well as in wine maturation [11]. More recently, PACs have also been proposed as viable alternatives for the replacement of synthetic food grade antioxidant [12,13] and antimicrobial [14] agents. In addition, PACs have also been reported for their beneficial properties for human health, more specifically, in the inhibition of enzymes related to high blood pressure [15] and carbohydrate metabolism [16,17,18], as well as anti-cancer [19,20] and anti-inflammatory activities [21].

The most common source of PACs for commercial use is Quebracho (*Schinopsis lorentzii*) heartwood which can have PACs contents up to 43% (w/w) [22]. Nevertheless, Quebracho trees are only present in South America and their use as raw material implies the transportation of the resulting extract across the globe. In addition, PACs obtained from Quebracho come from a dedicated crop, specifically grown for that purpose which is not the optimal use of water and arable land in terms of economic value since they can be found in high concentration in agroforestry by-products [23].

Agroforestry by-products could be used as alternative raw materials for the obtention of PACs which would allow for a more sustainable and efficient process as far as limited resources, such as water and arable land, are concerned. In addition, this approach is also valuable for the decrease of overall amount of waste as described by the European commission directive (2008/98/EC) [24] as well as to increase economic value of agroforestry by-products such as fruit peels [25] and wood barks [26] by taking advantage of their high PAC content as summarized elsewhere [23].

Grape pomace in particular, is a by-product of wine production which has been the subject of several dedicated reviews that explored its potential as a source of antimicrobial agents [27], human health promoter [28] and animal nutrition [29] in part due to its high PACs content.

Wine industry is one of the most important agricultural activities worldwide and produced 292 million hL of wine and 44 million tons of grape for that purpose in 2018 [30], which led to the production of approximately 11 million tons of grape pomace (considering that 1 kg of fresh grapes results in 0.25 kg of pomace). Currently, grape pomace has little to no commercial value and often represents additional costs for the producer related to their disposal.

Presently, the industrial use of PAC extracts is often limited by their high price, especially when compared to the available synthetic (yet more harmful) alternatives. In general, these extracts are obtained through the use of a hot pressurized sulfite aqueous solution [31]. Unfortunately, the amount of unextracted PACs can be as high as 62.5% in white grapes [32] and 62.3% in Norway spruce bark [33], resulting in a low yield process and a final extract that is mostly composed of PACs with lower degree of polymerization that are not as effective as the ones with higher degree of polymerization. Additionally, in order to achieve the intended mean degree of polymerization (mDP) further downstream processing is needed, making its final price prohibitive for most applications.

Therefore, to further increase the extraction efficiency and added value of PACs extracts it is essential to combine the use of by-products rich in these compounds with innovative extraction procedures. Some innovations come from the combination of ultrasound assisted or microwave assisted extraction with aqueous mixtures of organic solvents as discussed elsewhere [23]. Nevertheless, these approaches still rely on the use of harmful organic solvents and new greener alternatives must be found.

More recently, eutectic solvents (ESs) have been proposed as very promising media for the extraction of bioactive compounds from biomass [34]. ESs can be described as a mixture of two compounds (hydrogen bond acceptor (HBA) and hydrogen bond donor (HBD)) that have a decreased melting point when compared to the individual components and were first proposed as a solvent in the context of deep eutectic solvents (DES) by Abbot et al. [35]. These differentiate from ESs by the fact that the decrease in the melting point is greater than what would be expected in an ideal mixture [36]. (D)ESs are characterized by their biocompatibility, low toxicity, easiness of preparation and ability of being custom made to a specific application.

The application of ESs in the extraction of PACs is still limited, however they have been studied in the extraction of PACs from *Gingko biloba* leaves with satisfactory results [37]. A mixture of choline chloride and malonic acid at a molar proportion of 1:2 with 55% (m/m) of water at 65 °C was used and an improvement of 67% when compared with aqueous 70% (v/v) acetone was obtained.

Due to (D)ESs high viscosity, water addition is frequently employed, as shown in the example presented before [37]. Nevertheless, the use of ethanol for that purpose, a solvent that is naturally sourced and safe for human consumption, is still unexplored. In addition, it is also a more effective solvent at room temperature on the extraction of PACs [38] when compared to water and therefore, could represent a valuable option in the development of new solvent systems.

One aspect that is frequently overlooked in the extraction of PACs is the possibility of tuning the extract’s final characteristics, such as mDP and galloylation percentage (%Gal). mDP has been shown to positively correlate with the inhibition of α-glucosidase and pancreatic lipase [39], cellular antioxidant [40] and anti-inflammatory [21] activities and protein precipitation [41,42]. %Gal has been shown to influence antiviral activity [43] and protein precipitation [44] as well. In addition, both characteristics appear to play an important role in antiproliferation of human colon cancer cells [45].

Herein, the use of mixtures of ethanol, water and ESs in the obtention of PACs from white grape pomace was explored for the first time. Several combinations of HBAs and HBDs were screened and the best candidate was selected. The effects of mass fraction of HBA, HBD, water and ethanol were optimized using response surface methodology (RSM) to determine the best solvent composition in the tuning of the final extract characteristics. A similar approach was used to optimize the extraction conditions of PACs, namely the temperature, solid:liquid ratio and extraction time. PACs yield (*Y*_PAC_) was quantified using the acid butanol method and the mDP and %Gal were determined by phloroglucinolysis.

## 2. Materials and Methods 

### 2.1. Reagents and Equipment

All reagents and solvents were used as received without further processing. Acetone, concentrated hydrochloric acid, phloroglucinol, sodium sulfite, glycerol, ammonium iron (III) sulfate dodecahydrate and (+)-catechin hydrate were purchased from Sigma-Aldrich (St. Louis, MO, USA). Methanol, dichloromethane, absolute ethanol, 1-butanol, ascorbic acid and anhydrous sodium acetate were purchased from Fisher Scientific (Hampton, VA, USA). Choline chloride, betaine, proline and (D)-glucose were purchased from Acros Organics (Waltham, MA, USA). Urea and DL-malic acid were purchased from VWR (Radnor, PA, USA). Toyopearl HW-40S resin was acquired from Tosoh (Tokyo, Japan). HPLC-grade water, acetonitrile and formic acid were acquired from Fisher Scientific. 

The High-Performance Liquid Chromatography (HPLC) system was an Accela from Fisher Scientific with an Accela 600 LC pump and an Accela 80Hz Diode-Array detector (DAD). Chromatographic separation was carried out with a Hypersil GOLD C18 (2.1 × 100 mm with 1.9 µm particle size) column from Thermo Scientific. The MS system used for compound identification was a LCQ Fleet ion trap mass spectrometer from Thermo Finnigan with an electrospray ionization (ESI) source. Data acquisition was performed by using the Xcalibur data system from Thermo Finnigan (Waltham, MA, USA).

### 2.2. Grape Pomace Preparation

White grape pomace was obtained from a mixture of grape varieties from the Douro region in Portugal and was collected during the 2019 harvest after pressing for must extraction. The grape pomace was then kept frozen at −20 °C until freeze-drying after which it was kept tightly closed at room temperature protected from light until use.

### 2.3. Isolation of Grape Proanthocyanidins

PACs were isolated from grape pomace to use as standard for colorimetric quantification following the method described by Alwerdt et al. [46] with slight modifications. Briefly, freeze-dried grape pomace was firstly defatted with dichloromethane by Soxhlet extraction for 6 h. The defatted pomace was then extracted three times with aqueous 70% (v/v) acetone for 2 h at room temperature under continuous agitation at 100 rpm and with 10% (m/m) of biomass. After extraction, the supernatants were pooled together and the solvent was removed in a rotary evaporator at 40 °C. The crude dry residue was resuspended in a minimum amount of distilled water, freeze-dried and kept in a desiccator until further use. 2 g of the crude dry extract were dissolved in 20 mL of methanol and after vortexing thoroughly for 5 min, the suspension was centrifuged for 10 min at 6000 rpm. The supernatant was then loaded into a glass column with an internal diameter of 16 mm packed with 100 mm of Toyopearl HW-40 resin, previously equilibrated with methanol. The flow was kept at 1.5 mL/min with the extract being firstly washed with 300 mL of methanol followed by the elution of the fraction of interest with 250 mL of methanol with 30% (v/v) acetone. The solvent was completely removed from this fraction in a rotary evaporator at 40 °C, the dry residue was resuspended in a minimum amount of distilled water, freeze-dried and kept in a desiccator until further use.

### 2.4. Quantification of Proanthocyanidins Content by Acid Butanol Assay

PACs quantification was performed by the acid butanol assay as described by Porter et al. [47] with some modifications. Briefly, 82.5 µL of a methanolic solution containing a known amount of PACs extract was transferred into pressure and temperature resistant tubes and mixed with 500 µL of butanol reagent (butanol with 5% (v/v) of concentrated hydrochloric acid) and 18 µL of ammonium iron (III) sulfate dodecahydrate solution (20 mg/mL prepared in aqueous hydrochloric acid (2 M)). The mixture was incubated at 100 °C for 50 min and after cooling the absorbance at 520 nm was measured. The quantification was made using the purified PACs described previously as standard.

### 2.5. Determination of Proanthocyanidins Mean Degree of Polymerization and Galloylation Percentage by Phloroglucinolysis

The determinations of PAC’s mDP and %Gal were made following the Kennedy et al. [48] phloroglucinolysis method with slight modifications. Briefly, samples containing approximately 10 mg of PACs were dissolved in 1.0 mL of a freshly prepared methanol solution containing 50 g/L of phloroglucinol, 10 g/L of ascorbic acid and 0.1 M of hydrochloric acid. After spinning down for removal of insolubilized material, 400 µL of the mixtures were transferred to pressure resistant vials and were incubated at 50 °C for 1 h after which the reaction was stopped by adding 2 mL of 40 mM sodium acetate aqueous solution. The depolymerization products were quantified by HPLC with eluents A (acetonitrile with 0.1% (v/v) formic acid) and B (99% (v/v) water and 1% (v/v) of acetonitrile with 0.1% (v/v) of formic acid) and were filtered under vacuum with 0.2 µm pore before adding formic acid. The elution program started with 99% (v/v) of B for 3 min, decreased to 69% (v/v) over 27 min and to 0% (v/v) over 2 min, followed by an increase to 99% (v/v) over 4 min that were kept for 4 min. Peak identification was made by ESI-MS operated in the negative ion mode with operational conditions described elsewhere [49]. Quantification of phloroglucinolysis products was made with a calibration curve of (±)-catechin aqueous solution with concentrations ranging from 1 to 250 µg/mL. The mDP was calculated by dividing the sum of terminal and extension units by the sum of terminal units and the %Gal was calculated by dividing the sum of galloylated units by the sum of all units.

### 2.6. Extraction with Conventional Solvents

The efficiency of conventional solvents, namely, hot water (HWE), hot 2% (m/m) sodium sulfite solution (HSE), aqueous 70% (v/v) acetone (AAE), aqueous x% (m/m) ethanol (AEEx; *x* = 15, 30, 50, 70) was assayed in a single step solid-liquid extraction with 10% (m/m) of grape pomace. HWE and HSE were performed at 110 °C for 2 h while AAE and AEEx were performed at 30 °C for 4 h under continuous agitation at 100 rpm after which suspensions were centrifuged at 10,000 rpm for 10 min and the supernatants collected and stored at −20 °C until characterization.

### 2.7. Preparation of Mixtures of Eutectic Solvents and Water/Water:Ethanol

ESs mixtures were prepared by adding the appropriate mass of each component to a flask with a magnetic bar and placing it in a magnetic stirrer at room temperature or at 40 °C, if needed, until a continuous liquid phase was obtained.

### 2.8. Screening of Hydrogen Bond Acceptor and Hydrogen Bond Donor Combination

Different combinations of HBAs (choline chloride (ChCl), betaine (Bet), proline (Pro)) and HBDs (urea (Ur), malic acid (MalA), glucose (Glu) and glycerol (Glyc)) were screened at a 3:1 molar proportion, for 4 h at 30 °C with 10% (m/m) of grape pomace under continuous stirring at 100 rpm. The ES content was varied from 25% to 75% and the remainder was composed either by water or water:ethanol (1:1) mixture. After extraction, mixtures were centrifuged at 10,000× *g* rpm for 10 min, the supernatants were recovered and store at −20 °C until characterization.

### 2.9. Determination of Optimal Solvent Composition

After a preliminary selection of the most suitable ES, namely ChCl:Glyc and having verified the impact of the presence of water and ethanol, the optimal extraction media composition was determined using RSM for mixtures using D-optimal design in Expert Design v12 from StateEase (Minneapolis, MN, USA). The experimental design consisted of 20 experimental points in which the assayed variables were the mass fractions of choline chloride (*x*_ChCl_), glycerol (*x*_Glyc_), water (*x*_water_) and ethanol (*x*_EtOH_) and the experimental constraints were *x*_ChCl_ < 0.7, *x*_Glyc_ < 0.95, 0.05 < *x*_water_ < 0.5, *x*_EtOH_ < 0.4 and *x*_ChCl_ + *x*_EtOH_ < 0.7. Experimental solvent compositions are detailed in Table A1 and all extractions were performed at 30 °C under continuous agitation at 100 rpm with 10% (m/m) of grape pomace. Validation runs were performed in triplicate for the solvent compositions that resulted in the best *Y*_PAC_ and best %Gal.

### 2.10. Determination of Optimal Extraction Conditions

Optimal extraction conditions were determined using RSM with Box-Behnken Design in Expert Design v12 from StateEase (Minneapolis, MN, USA). The experimental design consisted of 15 experimental points in which the assayed variables were temperature (temp), biomass percentage (%BM) and extraction time (time) with minimum (−1) and maximum (1) values ranging from 70 to 110 °C, 5 to 20% (m/m) and 1 to 5 h, respectively. Experimental conditions for each run are detailed in Table A2. All extractions were performed under continuous agitation at 100 rpm and solvent composition was the one from which the highest *Y*_PAC_ values were obtained in the previous section. Validation runs were performed in triplicate for the experimental conditions that resulted in the best *Y*_PAC_ and best %Gal.

## 3. Results

### 3.1. Proanthocyanidins Extraction with Conventional Solvents

**Figure 2:**
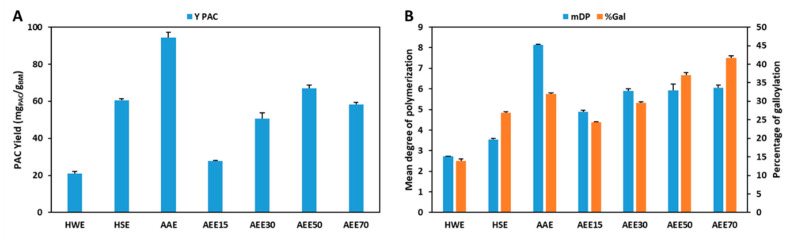
Comparison of *Y*_PAC_ (**A**) and mDP and %Gal (**B**) obtained with conventional solvents (HWE—hot water extraction; HSE—hot sulfite extraction; AAE—aqueous acetone extraction; AEEx—aqueous ethanol extraction).

The extraction yields obtained with the different conventional solvents, hot water extraction (HWE), hot sulfite extraction (HSE), aqueous acetone extraction (AAE) and aqueous ethanol extraction (AEEx) and the respective mDP and %Gal in a single step solid-liquid extraction are shown in Figure 2 and Table A3.

As expected, the best results were obtained in AAE as far as yield (94.4 mg_PAC_/g_BM_) and mDP (8.1) are concerned which further supports the use of aqueous acetone as a reference solvent for PACs extraction at a laboratory level. Despite being a very efficient extraction solvent, at a concentration of 70% (v/v) acetone it presents several risks for the operators and to the environment, requiring additional protective measures that represent increased operational costs.

Nevertheless, the results presented here also demonstrate that the use of ethanol:water mixtures with an ethanol content higher than 50% (m/m) enables the obtention of PACs extracts with higher %Gal (37.1%) when compared with the results obtained with AAE (31.9%). Furthermore, a direct correlation is verified between the ethanol content in the extraction mixture and %Gal that reaches its maximum value at 70% (m/m) ethanol with 41.6% of galloylated monomers. This trend was not verified for yield and mDP which show maximum values at 50% (m/m) ethanol (67.1 mg_PAC_/g_BM_) and a constant value above 30% (m/m) ethanol (5.9), respectively.

As far as the use of mixtures of ethanol and water is concerned, it is clear that despite the fact that the variation of ethanol allows a good control in the %Gal without affecting mDP considerably, this occurs in detriment of extraction yield for ethanol percentages higher than 50% (m/m) which indicates that AEE is not an efficient system by itself for the extraction of PACs.

In terms of organic solvent free extractions, the use of pure water with showed low yield (21.0 mg_PAC_/g_BM_), mDP (2.7) and %Gal (13.8%) which were improved with the addition of sodium sulfite, to values of 60.4 mg_PAC_/g_BM_, 3.5% and 26.9%, respectively. Nevertheless, although being possible to obtain comparable *Y*_PAC_, mDP and %Gal, these values are still considerably lower than the ones obtained with mixtures of organic solvents and water.

### 3.2. Screening of Hydrogen Bond Acceptor and Hydrogen Bond Donor Combination

HBA and HBD candidates were first selected based on their benign character and previous referencing in scientific literature for biomass processing [37,50,51,52]. Choline chloride (ChCl), betaine (Bet) and proline (Pro) were selected as HBAs while urea (Ur), malic acid (MalA), glucose (Glu) and glycerol (Glyc) were selected as HBDs. In a preliminary study (data not shown), it was concluded that mixtures containing Bet and Pro based ESs led to considerably lower extraction yields when compared to ChCl and therefore, their application in PACs extraction was not further explored.

**Figure 3:**
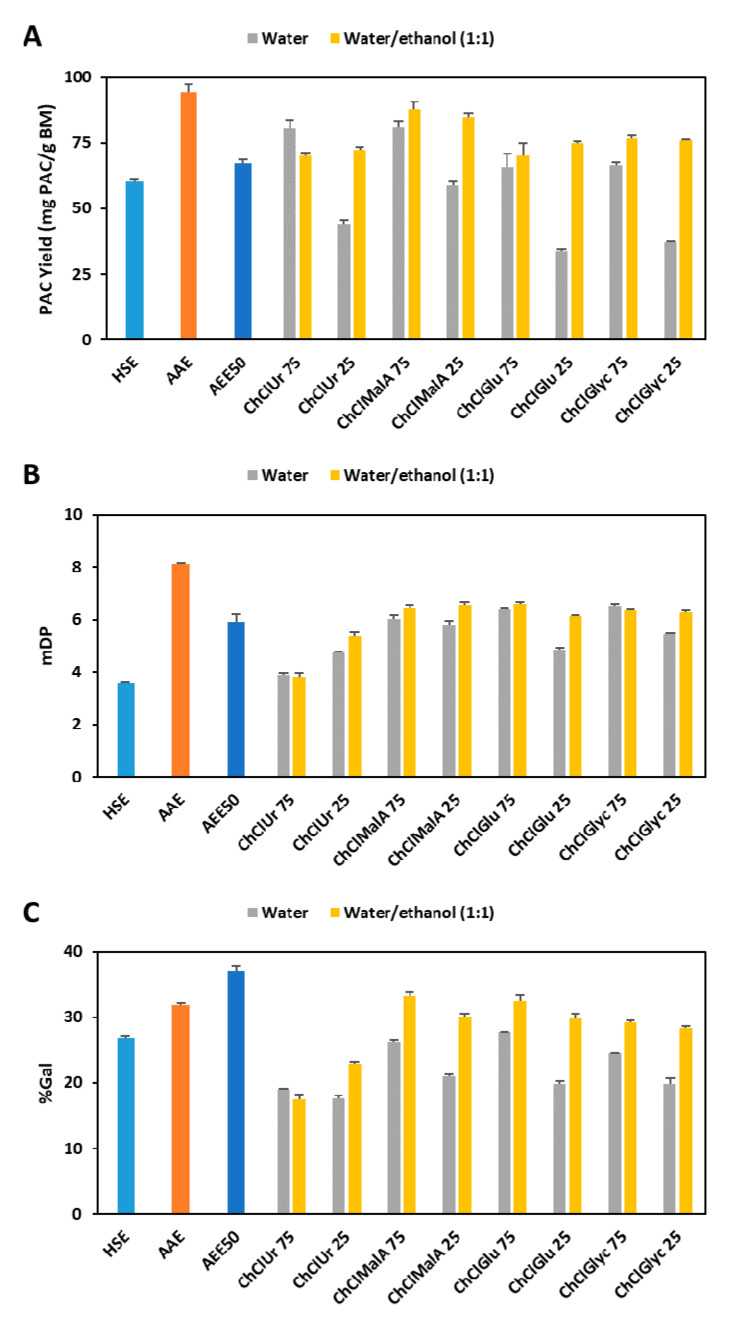
Comparison of *Y*_PAC_ (**A**), mDP (**B**) and %Gal (**C**) between conventional solvents (HSE, AAE and AEE50) and mixtures of ES with water or water and ethanol. (ChCl—choline chloride; Ur—urea; MalA—malic acid; Glu—glucose; Glyc—glycerol; the number following ES represents its percentage).

The effect of different HBDs in combination with ChCl on the *Y*_PAC_ is compared in Figure 3A and it is possible to observe that at a concentration of 75% of ES, the best results were obtained with Ur and MalA (80.7 and 81.4 mg_PAC_/g_BM_, respectively) followed by Glu and Glyc (65.9 and 66.4, respectively). Additionally, if the water content is increased to 75%, a decrease in *Y*_PAC_ of approximately 50% is observed for all candidates except for MalA which only decreases 30%. Nevertheless, this effect can be overcome if a mixture of water and ethanol is used instead of adding only water to ESs. This is especially true for ESs containing Glu and Glyc (75.0 and 76.2 mg_PAC_/g_BM_, respectively) which led to yields higher than the ones obtained with 75% ES and less accentuated for Ur and MalA (72.3 and 85.0 mg_PAC_/g_BM_, respectively).

In terms of mDP (Figure 3B), the best results were obtained with Glu and Glyc (6.4 and 6.5, respectively) followed by MalA (6.0) and Ur (3.9). As for *Y*_PAC_, the increase on the water content has a negative effect on the overall results, except for Ur and the addition of ethanol overcomes the reduction in the mDP. Similar observations were made for %Gal (Figure 3C) concerning the effect of HBD and water content. Nevertheless, the addition of ethanol has a more noticeable effect on the final result when compared to the effect on mDP which is corroborated by the results depicted in Figure 2B.

### 3.3. Determination of Optimal Solvent Composition

From the previous section, ChCl and Glyc mixtures were chosen as the best candidates for solvent composition optimization due to low price, chemical and microbiological stability. Additionally, water and ethanol content were also considered due to their effect on solvent viscosity reduction and %Gal content tuning, respectively.

Consequently, a 20-run mixture RSM experiment was performed where *x*_ChCl_, *x*_Glyc_, *x*_water_ and *x*_EtOH_ were varied as specified in Table A1 and *Y*_PAC_, mDP and %Gal were determined.

**Figure 4:**
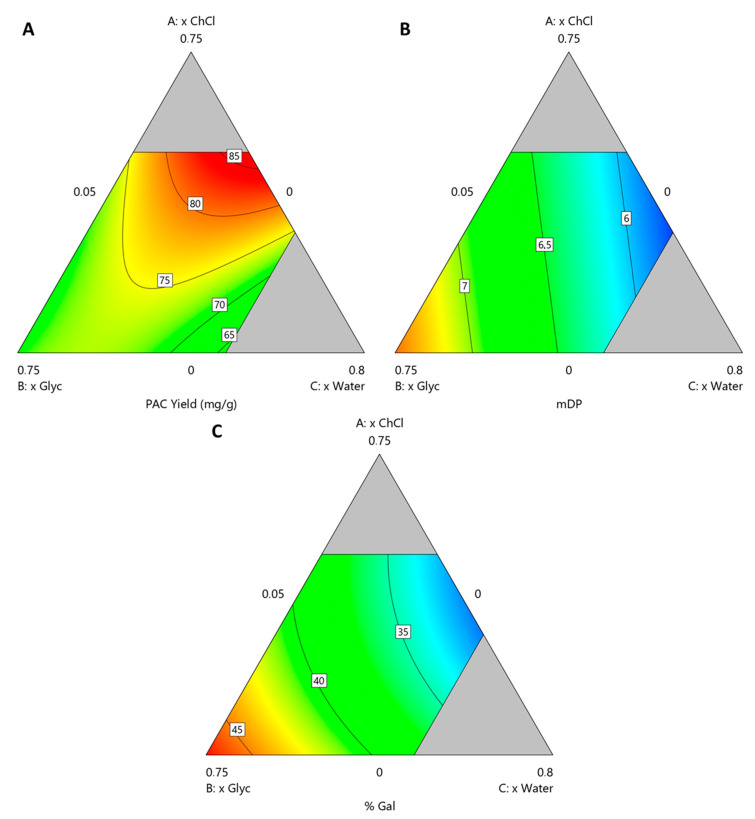
Contour plots obtained for solvent compostiion optimization at *x*_EtOH_ = 0.2 for (**A**) *Y*_PAC_, (**B**) mDP and (**C**) %Gal.

The resulting polynomials are presented in Equations (1)–(3) for *Y*_PAC_, mDP and %Gal, respectively, from which contour plots were derived and presented in Figure 4 where *x*_ChCl_, *x*_Glyc_ and *x*_water_ are varied and *x*_EtOH_ is kept constant at 0.2.

(1) $$\begin{array}{c} Y_{PAC}=72.2x_{ChCl}+58.3x_{Glyc}-13.8x_{water}+8.13x_{EtOH}-38.0x_{ChCl}x_{Glyc}\\ \;+145x_{ChCl}x_{water}+145x_{ChCl}x_{EtOH}+108x_{Glyc}x_{water}+129x_{Glyc}x_{EtOH}\\ \;+278x_{water}x_{EtOH} \end{array}$$

(2) $$mDP=6.21x_{ChCl}+7.87x_{Glyc}+4.49x_{water}+6.46x_{EtOH}+3.31x_{water}x_{EtOH}$$

(3) $$\begin{array}{c} \%Gal=39.1x_{ChCl}+46.3x_{Glyc}+22.7x_{water}+32.7x_{EtOH}-12.8x_{ChCl}x_{Glyc}-22.7x_{ChCl}x_{water}\\ \;+27.0x_{Glyc}x_{EtOH}+48.3x_{water}x_{EtOH} \end{array}$$

The performed ANOVA analysis revealed that the resulting models for *Y*_PAC_, mDP and %Gal all have *p*-values < 0.0001, adjusted *r*^2^ values of 0.92, 0.94 and 0.98, respectively and predicted *r*^2^ values of 0.82, 0.92 and 0.97, respectively, indicating the statistical robustness of the resulting quadratic models.

From these models it can be concluded that, with respect to *Y*_PAC_ (Figure 4A), *x*_ChCl_, *x*_water_ and *x*_EtOH_ have an optimal value at 0.5, 0.3 and 0.2, respectively and that *x*_Glyc_ has a detrimental effect on it. With this combination, the predicted values of *Y*_PAC_, mDP and %Gal are 86.6 mg_PAC_/g_BM_, 6.0 and 32.5%, respectively which represents 91.7% of the *Y*_PAC_ obtained with AAE, a decrease in mDP, 8.1 and a similar %Gal, 31.9%. The validation runs resulted in a *Y*_PAC_ of 72.4 mg_PAC_/g_BM_, mDP of 5.9 and %Gal of 30.6% which are in a reasonable agreement with the predicted values, validating the models. Based on the results obtained for ESs, it is possible to conclude that AAE is a more suitable method for laboratory scale analysis, especially if solvent removal is considered.

Nevertheless, when compared to the conventional extraction process, HSE, an increase in *Y*_PAC_ of 43.4% and higher mDP values, 6.0 in opposition to 3.5 is observed, which indicates the potential of the proposed system in replacing conventional methods on a purely *Y*_PAC_ basis.

If other aspects of the final extract are prioritized, namely mDP (Figure 4B) and %Gal (Figure 4C), with this system, it is possible to achieve, with this system, values that can go as high as 7.37 and 47.1%, respectively, while maintaining a *Y*_PAC_ of 72.2 mg_PAC_/g_BM_. This can be achieved with *x*_Glyc_, *x*_water_ and *x*_EtOH_ values of 0.68, 0.05 and 0.27, respectively, which represents an increase of 19.5% in *Y*_PAC_ when compared with the conventional extraction process, HSE. The validation runs resulted in a *Y*_PAC_ of 67.2 mg_PAC_/g_BM_, mDP of 7.5 and %Gal of 47.2% which are in a reasonable agreement with the values predicted by the model. This behavior is contrary to what would be expected, considering that PACs with a higher DP should be more difficult to extract and therefore an increase in *Y*_PAC_ should be associated with an increase in mDP which was not verified, reinforcing the idea that this solvent composition is more specific for PACs with higher DP.

### 3.4. Determination of Optimal Extraction Conditions

For the optimization of the extraction conditions, the solvent composition that resulted in a higher *Y*_PAC_ (*x*_ChCl_ = 0.5, *x*_water_ = 0.3 and *x*_EtOH_ = 0.2) was chosen since mDP and %Gal are still above what is obtained with HSE.

Consequently, a 15-run RSM experiment with Box-Behnken design was made where temperature, %BM and extraction time were varied as specified in Table A2 and *Y*_PAC_, mDP and %Gal were determined.

**Figure 5:**
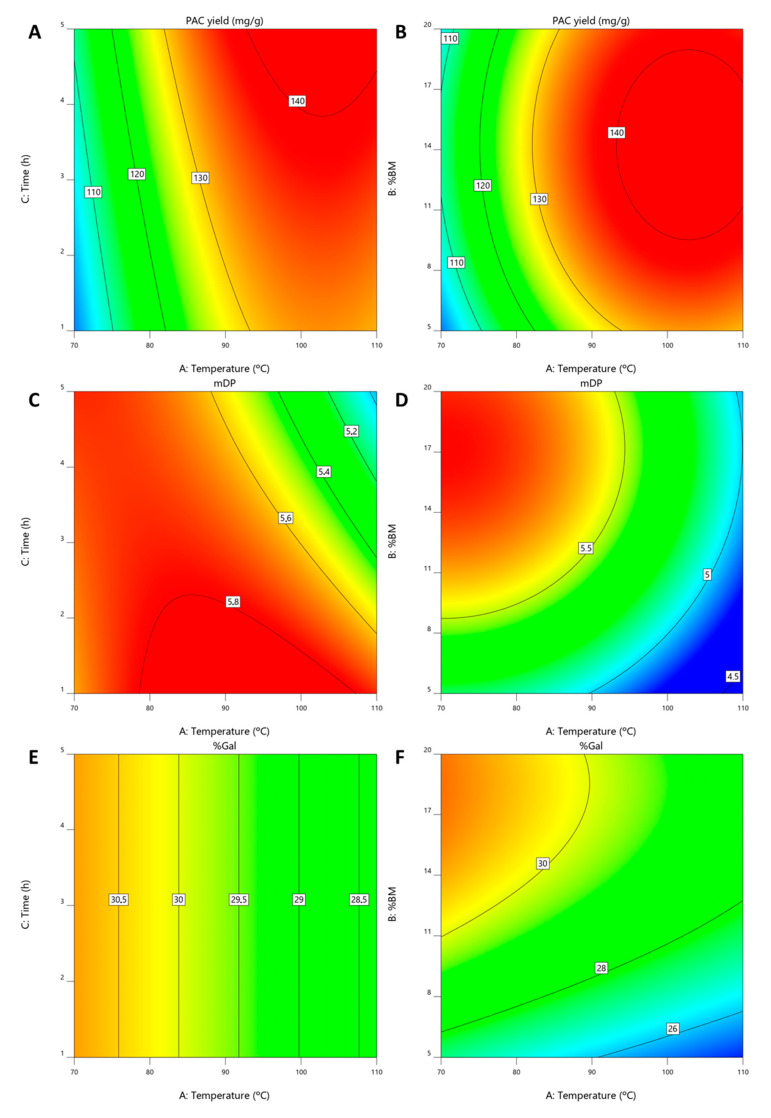
Contour plots obtained for extraction conditions optimization for (**A**,**B**) *Y*_PAC_, (**C**,**D**) mDP and (**E**,**F**) %Gal.

The resulting polynomials are presented in Equations (4)–(6) for *Y*_PAC_, mDP and %Gal, respectively, from which contour plots were derived and presented in Figure 5A–F where %BM was kept constant at 14.4% in A, C and E and extraction time was kept at 5 h in B, D and F.

(4) $$\begin{array}{c} Y_{PAC}=-184+6.10temp+1.81{\%}_{BM}-2.30time+0.338{\%}_{BM}\;time-0.0297temp^{2}\\ \;-0.122{\%}_{BM}^{2} \end{array}$$

(5) $$\begin{array}{c} mDP=-0.0559+0.0996temp+0.140{\%}_{BM}+0.430time-0.00571temp\;time\\ \;-0.000505temp^{2}-0.00407{\%}_{BM}^{2} \end{array}$$

(6) $$\%Gal=28.3-0.0629temp+0.794{\%}_{BM}-0.0214{\%}_{BM}^{2}$$

The performed ANOVA analysis revealed that the resulting models for *Y*_PAC_, mDP and %Gal have *p*-values of 0.0002, 0.0007 and <0.0001, respectively, adjusted *r*^2^ values of 0.89, 0.88 and 0.89, respectively and predicted *r*^2^ values of 0.73, 0.67 and 0.82, respectively, indicating the statistical robustness of the resulting quadratic models.

From these models, it can be concluded that the optimal *Y*_PAC_ values are obtained with 14.4% (m/m) of BM, at 102.8 °C for 5 h (Figure 5A,B). This resulted in predicted *Y*_PAC_ of 143.0 mg_PAC_/g_BM_, mDP of 5.2 and %Gal of 28.8% which despite representing a dramatic increase from what is obtained at 30 °C (65%) and with HSE (238%), also represents a decrease in mDP compared to the value obtained at 30 °C (6.0). The validation runs resulted in a *Y*_PAC_ of 144.1 mg_PAC_/g_BM_, mDP of 6.0 and %Gal of 28.3% which are in agreement with the model predictions.

In addition and even though the best results are obtained with 5 h of extraction time, this factor has a small impact on the overall *Y*_PAC_. Furthermore, if mDP is considered (Figure 5C) it becomes clear that at temperatures above 90 °C, the increase in extraction time causes a decrease in mDP, indicating thermal degradation of the PACs, which is in line with the results published by Ramos et al. [53].

With this in mind, the results can be improved considering both *Y*_PAC_ and mDP in which case the optimal conditions would be 99 °C with 13.4% of biomass for 1 h. In this situation the extraction process would have an *Y*_PAC_ of 133.6 mg_PAC_/g_BM_ and would result in a final extract with a mDP of 5.9 and %Gal of 28.8%. These conditions are not optimal for Y_PAC_ but would enable the obtention of an extract more similar to the one obtained at 30 °C in terms of mDP. The confirmation runs resulted in a *Y*_PAC_ of 125.9 mg_PAC_/g_BM_, mDP of 6.5 and %Gal of 29.6% which are in a reasonable agreement with what should be expected.

mDP also relates with the %BM (Figure 5D), being possible to obtain higher mDP values at higher biomass concentrations which further indicates the selectivity of the quaternary solvent system towards PACs with higher DP. This conclusion is based on the fact that with an increase in biomass concentration one should expected an increase in compounds that are more easily extracted in detriment of others, in this case PACs with higher DP.

As far as %Gal is concerned (Figure 5E,F), extraction time has no effect on the final result and, similarly to mDP, an increase in %Gal can be achieved with higher %BM and lower temperatures, although this effect is less accentuated.

## 4. Discussion

In order to select the better HBA–HBD combination for the intended application, ESs stability and possible interactions with PACs must also be considered.

More specifically, if only *Y*_PAC_ is considered, the combination of ChCl and MalA would be considered the best candidate, as shown in Figure 3A. Especially, considering that this specific mixture and others alike have been extensively characterized and proposed as valid alternatives for biomass processing [54]. Unfortunately and as described elsewhere ESs composed by ChCl and MalA are not long term stable at room temperature and are negatively affected by temperature even in short incubation times [55]. These would limit considerably not only the process optimization by means of increased temperature but the possibility of recycling the solvent which might be necessary in order to develop a feasible industrial process.

Despite resulting in *Y*_PAC_ that in some conditions are comparable to the ones obtained with MalA, ESs containing Ur give rise to extracts with low mDP and %Gal. In addition, contrary to what happens with the other candidates, Ur does not work synergistically with ethanol that as shown before is an important factor for %Gal tuning.

The use of Glu mixtures resulted in lower *Y*_PAC_ with little effect on mDP and %Gal. Nevertheless, Glu [56] and carbohydrate based [57] ESs have in general very high viscosity values which require the addition of considerable amounts of water in order to achieve reasonable viscosity levels. This in turn results, as discussed previously, in a reduction of the extraction efficiency and in a decrease in microbiological stability which makes solvent recycling not feasible.

The mixture of ChCl with Glyc presents comparable values of *Y*_PAC_ and mDP to the ones obtained with Glu mixtures but lower %Gal values. As in the case of MalA, Glyc-based solvents have been extensively described for biomass processing both in [51] and out [58] of the ES context. Additionally, Glyc is a cheap and abundant by-product obtained from biodiesel production that can be sustainably sourced [59] and is already largely used in food, medical and cosmetic industries due to its safety for human consumption. Recently, promising results were observed in the use of Glyc-based ES for the extraction of other flavonoids, namely apigenin, luteolin and quercetin from *Satureja thymbra* [60].

Considering the different mDP and %Gal that were obtained with different component proportions of the proposed quaternary system, it can be inferred that by varying ESs composition it is possible to tune the final extract characteristics to specific values of mDP and %Gal. More specifically, to values ranging from 5.95 to 7.37 for mDP and from 32.5 to 47.1% for %Gal, whilst maintaining an acceptable *Y*_PAC_. Additionally, if the temperature difference between the conditions used in the solvent composition optimization assay (30 °C) and conventional extraction method, HSE (110 °C), is considered a further increase in *Y*_PAC_ is to be expected.

The models developed here, in addition to allowing for the optimization of the process in terms of *Y*_PAC_, mDP and %Gal, can also be used to minimize compositional differences in the final extract that derive from differences in the grape variety used as raw material [61] or different plant parts from the same variety [62], enabling the compositional normalization of the final extract and facilitating its implementation in industrial processes. If taken to its full potential these models could also be employed to minimize differences between two completely different sources [23] further facilitating the industrial implementation of PAC extracts in the context of circular economy.

## 5. Conclusions

ESs have recently increased their popularity in biomass processing and the work here presented further demonstrates the potential that this type of solvents has on the improvement of conventional methodologies. More specifically, herein it is described for the first time, at the best of our knowledge, the possibility of selectively improving the final extracts’ content in PACs with higher degree of polymerization by means of a quaternary solvent system composed of choline chloride, glycerol, water and ethanol. Furthermore, it was shown that it is possible to tune the percentage of galloylated PACs to specific values by varying the mass fractions of the four components whilst not significantly compromising on the overall extraction yield.

In addition, the feasibility of using grape pomace as raw material for the extraction of PACs is further demonstrated reinforcing the role of by-products in the context of a circular economy.

## Abbreviations

PAC	proanthocyanidin
(D)ES	(deep) eutectic solvent
HBA	hydrogen bond acceptor
HBD	hydrogen bond donor
mDP	mean degree of polymerization
%Gal	galloylation percentage
HPLC	High-Performance Liquid Chromatography
DAD	Diode-Array Detector
HWE	hot water extraction
HSE	hot sulfite extraction
AAE	aqueous acetone extraction
AEE	aqueous ethanol extraction
BM	biomass
ChCl	choline chloride
Bet	betaine
Pro	proline
Ur	urea
MalA	malic acid
Glu	glucose
Glyc	Glycerol
RSM	Response Surface Methodology

## Appendix A

**Table A1:** Mass fractions of choline chloride, glycerol, water and ethanol used in optimization of solvent composition and respective measured and predicted values of *Y*_PAC_, mDP and %Gal.

Run	*x*_ChCl_	*x*_Glyc_	*x*_water_	*x*_EtOH_	*Y*_PAC_ (mg_PAC_/g_BM_)		mDP		%Gal	
					Measured	Predicted	Measured	Predicted	Measured	Predicted
1	0	0.1	0.5	0.4	69.6	68.3	6.2	6.3	39.7	39.8
2	0.263	0.316	0.05	0.371	75.7	74.5	6.5	6.8	40.7	40.9
3	0.237	0.446	0.317	0	59.7	60.9	6.5	6.4	33.6	34.1
4	0	0.5	0.5	0	48.7	49.2	6.3	6.2	34.8	34.5
5	0	0.1	0.5	0.4	66.8	68.3	6.3	6.3	39.9	39.8
6	0.54	0.25	0.05	0.16	72.5	74.2	6.8	6.6	38.3	38.2
7	0	0.5	0.5	0	50.6	49.2	6.2	6.2	34.9	34.5
8	0.3	0.04	0.26	0.4	84.1	84	6.5	6.3	36.5	36.1
9	0	0.526	0.282	0.192	73.7	72.4	7.0	6.8	42.9	42.4
10	0	0.95	0.05	0	57.4	59.8	7.7	7.7	44.9	45.1
11	0.7	0	0.3	0	81.3	76.9	5.7	5.7	29.1	29.4
12	0.104	0.699	0.05	0.147	68.5	68	7.2	7.4	43.9	44.5
13	0	0.55	0.05	0.4	72.2	71.5	7.2	7.2	47.3	46.6
14	0	0.95	0.05	0	61.5	59.8	7.5	7.7	44.5	45.1
15	0.401	0	0.5	0.099	72.4	71.5	5.6	5.5	28.9	28.1
16	0.378	0.552	0.07	0	60.5	58.6	7.0	7.0	39.4	38.7
17	0	0.55	0.05	0.4	69.7	71.5	7.4	7.2	46.5	46.6
18	0.147	0.231	0.5	0.122	61.9	63.2	5.7	6.0	31.8	33.4
19	0.459	0.235	0.306	0	65.5	66.7	6.0	6.1	31.6	31.2
20	0.7	0	0.3	0	73.3	76.9	5.7	5.7	29.3	29.4

*Y*_PAC_—proanthocyanidin yield, mDP—mean degree of polymerization, %Gal—galloylation percentage.

**Table A2:** Temperature, biomass percentage and time used in the optimization of extraction conditions and respective measured and predicted values of *Y*_PAC_, mDP and %Gal.

Run	Temp (°C)	%BM	Time (h)	*Y*_PAC_ (mg_PAC_/g_BM_)		mDP		%Gal	
				Measured	Predicted	Measured	Predicted	Measured	Predicted
1	90	20	1	111.9	116.5	5.8	5.9	30.6	29.9
2	90	12.5	3	131.8	133.8	5.8	5.7	28.8	29.2
3	90	12.5	3	134.0	133.8	5.5	5.7	29.0	29.2
4	90	5	5	130.4	127.4	5.1	5.0	26.0	26.6
5	110	20	3	130.4	128.7	5.4	5.4	28.2	28.7
6	90	20	5	133.1	134.3	5.7	5.6	29.0	29.9
7	110	12.5	5	133.6	141.0	4.8	4.9	28.4	27.9
8	70	12.5	1	102.3	102.9	5.7	5.6	30.5	30.5
9	70	12.5	5	113.2	110.6	5.7	5.7	30.4	30.5
10	70	20	3	102.4	98.3	5.7	5.7	32.0	31.2
11	90	5	1	129.5	129.8	5.3	5.3	27.1	26.1
12	70	5	3	95.6	101.6	5.1	5.1	26.6	27.3
13	90	12.5	3	138.7	133.8	5.6	5.7	28.7	29.2
14	110	5	3	135.2	131.9	4.8	4.8	24.5	24.8
15	110	12.5	1	135.8	133.3	5.7	5.7	28.3	27.9

*Y*_PAC_—proanthocyanidin yield, mDP—mean degree of polymerization, %Gal—galloylation percentage.

**Table A3:** Experimental values of *Y*_PAC_, mDP and %Gal obtained with conventional solvents.

Solvent	*Y*_PAC_ (mg_PAC_/g_BM_)	mDP	%Gal
HWE	11.5 ± 1.0	2.7 ± 0.01	13.8 ± 0.7
HSE	1.4 ± 0.1	3.5 ± 0.04	26.9 ± 0.3
AAE	91.5 ± 3.0	8.1 ± 0.04	31.9 ± 0.3
AEE15	18.9 ± 0.7	4.9 ± 0.08	24.4 ± 0.1
AEE30	39.7 ± 1.2	5.9 ± 0.08	29.6 ± 0.2
AEE50	46.7 ± 1.8	5.9 ± 0.30	37.0 ± 0.7
AEE70	45.3 ± 0.6	6.1 ± 0.12	41.6 ± 0.6

*Y*_PAC_—proanthocyanidin yield, mDP—mean degree of polymerization, %Gal—galloylation percentage; HWE—hot water extraction; HSE—hot sulfite extraction; AAE—aqueous acetone extraction; AEEx—aqueous ethanol extraction.
